# Relationship of metabolic syndrome and its components with -844 G/A and *HindIII *C/G *PAI-1 *gene polymorphisms in Mexican children

**DOI:** 10.1186/1471-2431-12-41

**Published:** 2012-03-29

**Authors:** Ulises De la Cruz-Mosso, José F Muñoz-Valle, Lorenzo Salgado-Goytia, Adrián García-Carreón, Berenice Illades-Aguiar, Eduardo Castañeda-Saucedo, Isela Parra-Rojas

**Affiliations:** 1 Unidad Académica de Ciencias Químico Biológicas, Universidad Autónoma de Guerrero, Avenida Lázaro Cárdenas S/N, Ciudad Universitaria, Chilpancingo, Guerrero CP. 39090, Mexico; 2 Grupo de Inmunogenética Funcional, Departamento de Biología Molecular y Genómica, Centro Universitario de Ciencias de la Salud, Universidad de Guadalajara, Jalisco, Mexico

**Keywords:** Metabolic syndrome, PAI-1, Polymorphism, Dyslipidemia, Children

## Abstract

**Background:**

Several association studies have shown that -844 G/A and *HindIII *C/G *PAI-1 *polymorphisms are related with increase of PAI-1 levels, obesity, insulin resistance, glucose intolerance, hypertension and dyslipidemia, which are components of metabolic syndrome. The aim of this study was to analyze the allele and genotype frequencies of these polymorphisms in *PAI-1 *gene and its association with metabolic syndrome and its components in a sample of Mexican mestizo children.

**Methods:**

This study included 100 children with an age range between 6-11 years divided in two groups: a) 48 children diagnosed with metabolic syndrome and b) 52 children metabolically healthy without any clinical and biochemical alteration. Metabolic syndrome was defined as the presence of three or more of the following criteria: fasting glucose levels ≥ 100 mg/dL, triglycerides ≥ 150 mg/dL, HDL-cholesterol < 40 mg/dL, obesity BMI ≥ 95^th ^percentile, systolic blood pressure (SBP) and diastolic blood pressure (DBP) ≥ 95^th ^percentile and insulin resistance HOMA-IR ≥ 2.4. The -844 G/A and *HindIII *C/G *PAI-1 *polymorphisms were analyzed by PCR-RFLP.

**Results:**

For the -844 G/A polymorphism, the G/A genotype (OR = 2.79; 95% CI, 1.11-7.08; *p *= 0.015) and the A allele (OR = 2.2; 95% CI, 1.10-4.43; *p *= 0.015) were associated with metabolic syndrome. The -844 G/A and A/A genotypes were associated with increase in plasma triglycerides levels (OR = 2.6; 95% CI, 1.16 to 6.04; *p *= 0.02), decrease in plasma HDL-cholesterol levels (OR = 2.4; 95% CI, 1.06 to 5.42; *p *= 0.03) and obesity (OR = 2.6; 95% CI, 1.17-5.92; *p *= 0.01). The C/G and G/G genotypes of the *HindIII *C/G polymorphism contributed to a significant increase in plasma total cholesterol levels (179 vs. 165 mg/dL; *p *= 0.02) in comparison with C/C genotype.

**Conclusions:**

The -844 G/A *PAI-1 *polymorphism is related with the risk of developing metabolic syndrome, obesity and atherogenic dyslipidemia, and the *HindIII *C/G *PAI-1 *polymorphism was associated with the increase of total cholesterol levels in Mexican children.

## Background

Metabolic syndrome (MetS) is a common disorder caused by a combination of poor diet, sedentary lifestyle and genetic predisposition [1], the presence of MetS in children is the main risk factor that predisposes to the development of cardiovascular and metabolic diseases such as atherosclerosis and type 2 diabetes mellitus in adulthood [2]. The components of MetS include obesity, insulin resistance, hyperglycemia, atherogenic dyslipidemia, and hypertension [1,3,4]. Besides these components, a decrease in fibrinolytic capacity has been shown to contribute to the development of this syndrome, which has been generally attributed to increased levels of plasminogen activator inhibitor-1 (PAI-1) [5,6].

PAI-1 is the main inhibitor in the plasminogen activation system (PAS), which comprises an inactive proenzyme, plasminogen, which can be converted into its active form plasmin by the action of physiological plasminogen activators (PAs). PAs degrades fibrin into soluble products, being PAI-1 one of the main regulators of fibrinolysis [7]. Increased PAI-1 levels in plasma are associated with the development of myocardial infarction and the formation/progression of chronic inflammatory diseases such as atherosclerosis and cardiovascular disease [8,9]. Increased of PAI-1 levels also have been linked with risk factors such as obesity, insulin resistance, glucose intolerance, hypertension and dyslipidemia (low HDL plasma levels and hypertriglyceridemia), which together are components of metabolic syndrome [10-13].

The human *PAI-1 *gene is ~ 12.2 kb long, contains nine exons and 8 introns, and is located on chromosome 7q22. To date about 180 single nucleotide polymorphisms (SNP) in the PAI-1 gene have been described [14,15]. Association studies have shown that polymorphisms located in the promoter region of *PAI-1 *gene shows a relationship with the concentrations of lipids (low HDL) in Mexico-American population [16,17]. One of the polymorphisms in the promoter *PAI-1 *gene is the -844 G/A polymorphism, which has been associated with risk factors such as increased plasma levels of PAI-1, glucose, insulin resistance, triglycerides and low HDL, as well as with several diseases including deep vein thrombosis, coronary artery disease, rheumatoid arthritis and systemic lupus erithematosus [18-21]. Other SNP that is interesting is the *HindIII *C/G polymorphism located in the 3' untranslated region (UTR) of *PAI-1 *gene, which has been related to high levels of cholesterol and insulin in myocardial infarction patients [22].

Based on this knowledge, both *PAI-1 *polymorphisms are good candidates that might contribute to the pathological features associated to the MetS. Therefore, we designed this study to analyze allele and genotype frequencies of -844 G/A and *HindIII *C/G *PAI-1 *polymorphisms and its association with MetS and its components in a sample of Mexican Mestizo children.

## Methods

### Patients and healthy subjects

All children enrolled in the study were of Mexican Mestizo population born in the State of Guerrero, Mexico, with a family history of ancestors, at least back to the third generation born in our State. This cross-sectional study was carried out between June and December 2008. Participants were recruited of three schools in the urban area from Chilpancingo, Guerrero, Mexico. The total group included 100 children with age range 6-11 years, divided in two groups: a) 48 children diagnosed with MetS and b) 52 children metabolically healthy without any clinical or biochemical alteration. The children with one or two clinic or metabolic alterations were excluded.

Informed written consent was obtained from all parents or guardians before enrollment of children in the study. Approval for the study was obtained from the Research Ethics Committee of the University of Guerrero according to the ethical guidelines of 2008 Declaration of Helsinki.

### Clinical and anthropometric measurements

Body weight was determined using a Tanita body composition monitor (Tanita BC-553, Arlington, USA) and height was measured to the nearest 0.1 cm using a stadiometer (Seca, Hamburg, Germany). From these measurements, body mass index was calculated (BMI = weight/height^2^, kg/m^2^). The circumferences were measured by duplicate using a diameter tape accurate to within ± 0.1 cm (Seca 201, Hamburg, Germany). Waist circumference was measured at the level of the umbilicus and the superior iliac crest. The measurement was made at the end of a normal expiration while the subjects stood upright, with feet together and arms hanging freely at the sides. Hip circumference was measured at the maximum point below the waist, without compressing the skin. The waist-to-hip ratio was calculated as waist/hip. The thickness of four skinfolds was measured to the nearest 0.1 mm, in duplicate, using skinfold caliper (Dynatronics Co, Salt Lake City, USA): triceps, biceps, subscapular and suprailiac. The duplicate measures were averaged.

Blood pressure (BP) was measured on the right arm of children seated and a rest for at least 5 min. Two consecutive measures were obtained at 1-min interval with an aneroid sphygmomanometer (Riester CE 0124, Jungingen, Germany). Hypertension was defined as the average of the two measurements where the systolic BP (SBP) or diastolic BP (DBP) is ≥ 95th percentile for age and gender was determined [23]. The classification of obesity was made using the 2000 Center for Disease Control and Prevention growth charts defined as normal weight 5^th^-85^th ^percentiles and obesity ≥ 95th percentile [24].

### Biochemical measurements and definitions

A blood sample was obtained from each child from antecubital venipuncture after overnight fast. Total serum cholesterol, triglycerides, HDL-cholesterol, LDL-cholesterol and glucose levels were obtained using a semi-automated equipment (COBAS MIRA), insulin levels were determined by immunoenzymatic assay (GenWay INS-EASIA kit).

The homeostasis model assessment of insulin resistance (HOMA-IR score) was used to determine insulin resistance in children; this score was calculated with the following formula: fasting serum insulin (μU/mL) × fasting plasma glucose (mmol/L)/22.5 taking scores ≥ 75^th ^percentile (HOMA-IR ≥ 2.4) as the presence of insulin resistance.

We employed International Diabetes Federation proposal for metabolic syndrome definition in children aged 10-16 years old for blood glucose and lipid levels [25]. In this study, MetS was defined as the presence of three or more of the following criteria: fasting glucose levels ≥ 100 mg/dL, triglycerides ≥ 150 mg/dL, HDL-cholesterol < 40 mg/dL, obesity BMI ≥ 95^th ^percentile, SBP and DBP ≥ 95^th ^percentile and insulin resistance HOMA-IR ≥ 2.4.

### Genotyping of -844 G/A and *HindIII *C/G *PAI-1 *polymorphism

The genomic DNA (gDNA) was isolated from peripheral blood leukocyte according to the salting out method [26]. The -844 G/A and *HindIII *C/G *PAI-1 *single nucleotide polymorphisms were analyzed by polymerase chain reaction-restriction fragment length polymorphism (PCR-RFLP). Amplification of -844 *PAI-1 *promoter region was done in a thermal cycler (Techne, TC-312, Cambridge, UK) using the following oligonucleotides: 5'CAGGCTCCCACTGATTCTAC3' (Forward) and 5'GAGGGCTCTCTTGTGTCAAC3' (Reverse) [27]. PCR was carried out in a final volume of 20 μL containing 1 μg of gDNA, 20 μM of each oligonucleotide, 1.25 U/μL *Taq *DNA polymerase, supplied buffer enzyme 1×, MgCl_2 _2.5 mM, and 2.5 mM of each deoxynucleotide triphosphate (dNTP) (Invitrogen Life Technologies, Carlsbad, Ca). PCR reaction was performed by initial denaturation at 94°C for 3 min, 30 cycles of amplification at 94°C for 30 seconds for denaturation, 60°C for 30 seconds for annealing, and 72°C for 30 seconds for extension. Finally, 72°C for 1 min was used for ending extension, resulting in a 510 base pair amplified fragment analyzed on a 6% polyacrylamide gel stained with silver nitrate. Amplified fragments of -844 *PAI-1 *polymorphism were digested for 1 hour at 37°C with 3 U of *XhoI *(New England Biolabs, Beverly, Mass.) restriction enzyme. Afterward, restriction fragments were analyzed by electrophoresis on a 6% polyacrylamide gel stained with silver nitrate. The G/G wild-type genotype was digested and appeared as 364 and 146 bp fragments, whereas the A/A polymorphic genotype (absence of the *XhoI *site) migrated as a 510 bp fragment.

The *HindIII *polymorphism was detected using the following oligonucleotides: 5'GCCTCCAGCTACCGTTATTGTACA3' (Forward) and 5'CAGCCTAAACAACAGAGACCCC3' (Reverse) [28]. PCR was carried out in a final volume of 20 μL containing 1 μg of gDNA, 3 μM of each oligonucleotide, 1.25 U/μL *Taq *DNA polymerase, supplied buffer enzyme 1×, MgCl_2 _1.5 mM, and 2.5 mM of each dNTP (Invitrogen Life Technologies). PCR reaction was performed by initial denaturation at 94°C for 3 min, 30 cycles of amplification at 94°C for 30 seconds for denaturation, 60°C for 30 seconds for annealing, and 72°C for 30 seconds for extension. Finally, 72°C for 1 min was used for ending extension, resulting in a 755 bp amplified fragment analyzed on a 6% polyacrylamide gel stained with silver nitrate. Amplified fragments of Hind III *PAI-1 *polymorphism were digested for 1 hour at 37°C with 5 U of *HindIII *(New England Biolabs) restriction enzyme. Afterward, restriction fragments were analyzed by electrophoresis on 6% polyacrylamide gel stained with silver nitrate. The C/C wild-type genotype was digested and appeared as 567 and 188 bp fragments, whereas the G/G polymorphic genotype (absence of *HindIII *site) migrated as a 755 bp fragment. To confirm the results, genotyping of both polymorphisms were done in duplicate in all cases and were randomly selected only a few -844 and *HindIII PAI-1 *genotypes for sequencing.

### Statistical analysis

Statistical analysis was performed using the statistical software STATA v 9.2. For the descriptive analysis, nominal variables were expressed as frequencies, continuous variables normally distributed as mean and standard deviation, and those not normally distributed were expressed as medians and percentile 5 and 95. We determined genotype and allele frequencies for the polymorphisms -844 and *HindIII PAI-1 *gene by direct counting, we performed chi-square test to compare proportions between groups (MetS vs. metabolically healthy) and to evaluate the Hardy-Weinberg equilibrium. The linkage disequilibrium between both SNPs was determined as D'.

The significance of the differences between the biochemical and anthropometric parameters by genotypes (G/G vs. GA + AA -844 and C/C vs. C/G + G/G *HindIII PAI-1*) was determined using student *t *test and by Mann Whitney. To evaluate the effect of polymorphism we used models of linear and logistic regression adjusted by gender and age. Differences were considered statistically significant at *p *< 0.05.

## Results

The present study included 100 Mexican Mestizo children of both genders, aged 6 to 11 years. Children were classified into two groups made up of 52 children metabolically healthy and 48 children with MetS, according the criteria mentioned above. The prevalence of components de MetS in the cases group was: 33.04% for obesity + hyperglycemia + high triglycerides, 29.79% for obesity + hyperglycemia + high triglycerides + low HDL-cholesterol, 14.89% for obesity + hyperglycemia + low HDL-cholesterol and others combinations with minor prevalence (data no shown).

In this study, both polymorphisms evaluated were in Hardy-Weinberg equilibrium (*χ*^2 ^= 0.005; *p *= 1.0 for -844 G/A polymorphism, and *χ*^2 ^= 0.62; *p *= 0.66 for *HindIII *C/G polymorphism). The linkage disequilibrium (D') between both SNPs was 0.81. The distribution of allele and genotype frequencies between the two groups did not show significant differences for *HindIII *C/G polymorphism, but for the -844 G/A polymorphism we observed a significant differences in genotype (*p *= 0.034) and allele frequencies (*p *= 0.015) between the two groups, with an OR of 2.79 (95% CI, 1.11 to 7.08) for the G/A genotype and an OR of 2.2 (95% CI, 1.10 to 4.43) for A allele, which indicates that children who were carriers of the risk A allele, have 2.2 fold more susceptibility to present MetS, and in children carrying the G/A genotype the risk increases to 2.79 fold (Table 1).

**Table 1:** Genotype and allele frequencies of -844 and *HindIII PAI-1 *polymorphisms in cases and controls

Polymorphism	Metabolic syndrome % (*n *= 48)	Metabolically healthy % (n = 52)	P value*	OR CI (95%); *p *value
**-844 G/A *PAI-1***				

**Genotype**				
**G/G^§^**	40 (19)	65 (34)	0.034	1.0
**G/A**	52 (25)	31 (16)		2.8 (1.11-7.08); 0.015
**A/A**	8 (4)	4 (2)		3.6 (0.45-42.06); 0.15

**Allele**				
**G**	66 (63)	81 (84)	0.015	1.0
**A**	34 (33)	19 (20)		2.2 (1.10-4.43); 0.015

**Genetic model**				

**Do**				
**G/G^§^**	40 (19)	65 (34)	0.01	1.0
**G/A + A/A**	60 (29)	35 (18)		2.9 (1.18-7.05); 0.009

***HindIII *C/G *PAI-1***				

**Genotype**				
**C/C^§^**	50 (24)	63 (33)	0.28	1.0
**C/G**	44 (21)	35 (18)		1.6 (0.65-3.95); 0.25
**G/G**	6 (3)	2 (1)		4.12 (0.30-222.9); 0.22

**Allele**				
**C**	72 (69)	81 (84)	0.13	1.0
**G**	28 (27)	19 (20)		1.64 (0.8-3.36); 0.13

**Genetic model**				

**Do**				
**C/C^§^**	50 (24)	63 (33)	0.17	1.0
**C/G + G/G**	50 (24)	37 (19)		1.73 (0.75-4.17); 0.13

*Chi square test *χ*^2^; OR = odds ratio; CI = confidence interval; Do = dominant model

**^§^**Reference category

Demographic, clinical and biochemical variables were compared by gender in all children. Only was observed a difference, the boys had higher fasting glucose levels than girls (median, 97 vs. 93 mg/dL; *p *= 0.04) (Table 2).

**Table 2:** General characteristics by gender in all children

	Gender		
**Variables**	**Boys** ***n *= 48**	**Girls** ***n *= 52**	***p *value**

**Age (years)^c^**	9 (6-11)	9 (6-11)	0.1

**BMI (kg/m^2^)^c^**	18 (14-28)	18 (14-26)	0.1

**Obesity (%)**			0.5

**Yes**	24 (50)	23 (44)	

**No**	24 (50)	29 (56)	

**Circumferences**			

**Waist (cm)^c^**	67 (55-90)	66 (56-85)	0.2

**Arm (cm)^c^**	21 (17-28)	20 (16-29)	0.4

**Skinfolds**			

**Biceps (mm)^b^**	15 ± 4.7	16 ± 4.4	0.2

**Triceps (mm)^c^**	14 (10-22)	15(9-22)	0.5

**Subscapular (mm)^c^**	13 (6-22)	15(6-22)	0.4

**Biochemical measurements**			

**Glucose (mg/dL)^c^**	97 (75-112)	93(70-112)	0.04

**Total cholesterol (mg/dL)^b^**	175 ± 30	168 ± 29	0.2

**Triglycerides (mg/dL)^c^**	101(36-204)	102 (38-200)	0.8

**HDL-C (mg/dL)^c^**	43(25-98)	46 (23-99)	0.9

**Insulin (μU/mL)^c^**	6 (0.8-31.3)	8 (0.4-21)	0.8

**HOMA^c^**	1.5 (0.18-7.2)	1.7 (0.08-5.1)	0.6

**Insulin resistance**^a^			0.07

**Yes**	13 (41)	15 (37)	

**No**	19 (59)	25 (63)	

a) Data provided in *n *and percentages. Chi square test; b) Data provided in means ± SD. Student's *t *test; c) Data provided in median and percentile 5-95. Mann-Whitney test

Demographic, clinical and biochemical variables were compared by genotypes of both *PAI-1 *polymorphisms according to a dominant genetic model. For the -844 G/A polymorphism the G/A and A/A genotypes were grouped for this genetic model, the -844 G/A + A/A group showed a high prevalence of obesity (60%; *p *= 0.01), an increase in thickness of the biceps (16 mm; *p *= 0.05), triceps (16 mm; *p *= 0.01) and subscapular skinfolds (15 mm; *p *= 0.03) and arm circumference (22 cm; *p *= 0.04), as well as decrease in HDL levels (39 mg/dL; *p *= 0.04) in comparison with G/G group (Table 3). To estimate in all children, the association of -844 G/A + A/A genotypes with demographic, clinical and biochemical variables that showed significant differences or tendencies, we used logistic regression models adjusted by age and gender. The -844 G/A + A/A genotypes were associated with increase in plasma triglycerides levels (OR = 2.6; 95% CI, 1.16 to 6.04; *p *= 0.02), decrease in plasma HDL-cholesterol levels (OR = 2.4; 95% CI, 1.06 to 5.42; *p *= 0.03) and obesity (OR = 2.6; 95% CI, 1.17-5.92; *p *= 0.01) (Table 4). However, we did not find a relationship with biceps, triceps and subscapular skinfolds as well as arm circumference (data no shown).

**Table 3:** General characteristics according *PAI-1 *polymorphism following a dominant genetic model

	-844 G/A *PAI-1*			*HindIII *C/G *PAI-1*		
**Variables**	**G/G** ***n *= 53**	**G/A + A/A** ***n *= 47**	***p *value**	**C/C** ***n *= 57**	**C/G + G/G** ***n *= 43**	***p *value**

**Age (years)^c^**	9 (6-11)	9 (6-11)	0.22	9 (6-11)	9 (6-11)	0.08

**Gender (%)^a^**			0.30			0.06

**Male**	28 (53)	20 (43)		32 (56)	16 (37)	

**Female**	25 (47)	27 (57)		25 (44)	27 (63)	

**BMI (kg/m^2^)^c^**	17 (13.7-26.6)	20 (15.1-27.5)	0.09	18 (14-29)	20 (15-26)	0.67

**Obesity (%)**			0.01			0.25

**Yes**	19 (36)	28 (60)		24 (42)	23 (53)	

**No**	34 (64)	19 (40)		33 (58)	20 (47)	

**Circumferences**						

**Waist (cm)^c^**	66 (54-87)	69 (57-89)	0.22	67 (54-90)	66 (57-88)	0.84

**Arm (cm)^c^**	20 (16-28)	22 (17-29)	0.04	21 (16-30)	21 (17-28)	0.68

**Skinfolds**						

**Biceps (mm)^b^**	15 ± 4.32	16 ± 4.70	0.05	15 ± 4.2	16 ± 4.9	0.27

**Triceps (mm)^c^**	13 (9-22)	16 (10-20.5)	0.01	14 (9.5-21.5)	15 (9-22)	0.75

**Subscapular (mm)^c^**	13 (5.5-22)	15 (8.5-22)	0.03	14 (6-25)	13 (6-22)	0.85

**Biochemical measurements**						

**Glucose (mg/dL)^c^**	95 (72-116)	96 (72-112)	0.38	95 (72-110)	95 (72-112)	0.59

**Total cholesterol (mg/dL)^b^**	168 ± 27.91	175 ± 31.05	0.20	165 ± 29.6	179 ± 28	0.02

**Triglycerides (mg/dL)^c^**	86 (38-225)	150 (36-200)	0.09	91 (29-200)	116 (38-204)	0.13

**HDL-C (mg/dL)^c^**	53 (25-102)	39 (23-89)	0.04	49 (21-99)	40 (25-98)	0.54

**Insulin (μU/mL)^c^**	5 (0.09-27.5)	10 (1.25-22.7)	0.10	7 (0.7-25)	7 (0.5-23)	0.91

**HOMA^c^**	0.5 (0-6.04)	1 (0-5.5)	0.30	1.62 (0.1-6.0)	1.8 (0.1-6.0)	0.89

**Insulin resistance^a^**			0.19			0.78

**Si**	9 (17)	13 (28)		12 (29)	10 (32)	

**No**	44 (83)	34 (72)		29 (71)	21 (68)	

a) Data provided in *n *and percentages. Chi square test; b) Data provided in means ± SD. Student's *t *test; c) Data provided in median and percentile 5-95. Mann-Whitney test

**Table 4:** Association of G/A + A/A genotypes of -844 *PAI-1 *polymorphism with obesity and lipid levels

-844 *PAI-1* Genotypes	Obesity		OR	(95% CI)	p* value
				
	< 95^th ^percentile % (*n *= 58)	≥ 95^th ^percentile % (*n *= 42)			
**G/G^§^**	64 (34)	40 (19)	1		

**G/A + A/A**	36 (19)	60 (28)	2.6	1.17-5.92	0.01

	**Plasma triglycerides levels**				
				
	**< 150 mg/dL % (*n *= 61)**	**> 150 mg/dL % (*n *= 39)**			

**G/G^§^**	62 (38)	38 (15)	1		

**G/A + A/A**	38 (23)	62 (24)	2.6	1.16-6.04	0.02

	**Plasma HDL-cholesterol levels**				
				
	**> 40 mg/dL % (*n *= 58)**	**< 40 mg/dL % (*n *= 42)**			

**G/G^§^**	68 (36)	32 (17)	1		

**G/A + A/A**	47 (22)	53 (25)	2.4	1.06-5.42	0.03

*Chi square test, **^§ ^**Reference category, OR = odds ratio, CI = confidence interval

For the *HindIII *C/G polymorphism, when the C/G and G/G genotypes were grouped, the C/G + G/G group showed only an increase in plasma total cholesterol levels (179 mg/dL; *p *= 0.02) in comparison with C/C group (165 mg/dL).

## Discussion

This study shows the association of two polymorphisms in the *PAI-1 *gene with the development of MetS and its components such as obesity and atherogenic dyslipidemia in a Mexican children population.

Regarding the distribution of genotype and allele frequencies of both polymorphisms, for the *HindIII *C/G polymorphism we found a high frequency of C allele, similar to previous reports in Mexican mestizo population and Caucasian population, however the G allele frequency was lower in Mexican population [18,21,29]. On the other hand, the -844 G/A polymorphism we observed that is distributed inversely to those reported in Caucasian populations, in which the A allele is more frequent than the G allele [12,18,19]. In our study this polymorphism had a high frequency of G allele and a lower frequency of A allele, consistent with already reported frequencies in previous studies in Mexican mestizo population [20,30], suggesting that in the Mexican population there is a high frequency of allele G.

According to our results, the differences observed in the distribution of -844 G/A polymorphism may be attributed to the racial influence, which is central to the heterogeneous distribution of genetic polymorphisms. It is known that the Mexican population originated from a mixture of European (4.2 to 70.8%) and African (0.9 to 40.5%) populations with Amerindian groups (27.6 to 94.5%), giving origin to the Mexican mestizo population, which has a higher genetic diversity in the distribution of this and other polymorphisms [31]. This can explain the differences in the distribution of genotypic and allelic frequencies of our population with other populations in the world.

As an important finding, in our study we found significant differences in the distribution of genotype and allele frequencies of -844 G/A polymorphism in both groups, determining an OR of 2.2 for A allele, and an OR of 2.79 for G/A genotype, which indicates that children who carry the A allele are 2.2 fold more susceptible to develop MetS and children who are carriers of the G/A genotype have a 2.79 fold increased risk of developing the syndrome, compared to those who are carriers of G allele and G/G genotype. These results obtained in our study are similar to those reported in a previous study done in Caucasian population in which A/A genotype was associated with the susceptibility of developing MetS (OR, 4.87; *p *< 0.001) [12]. These consistent results reported in different populations may be due to the effect of the polymorphism on the levels of the protein, since it has been reported that the base change of G to A at position -844 of the promoter *PAI-1 *gene generates a binding site consensus sequence for Ets nuclear protein, which could be involved in regulating gene expression and influencing the increase in PAI-1 plasma protein levels [32,33]. While for the *HindIII *C/G polymorphism not significant differences were found in genotype and allele distribution, but it has been reported that the base change of C to G at the 3' UTR of *PAI-1 *gene might plays an important role in the disruption of the translational regulation process and cause changes in the translational levels of messenger ribonucleic acid (mRNA) in both physiological and pathological conditions, resulting in an increase in PAI-1 plasma protein levels [34].

We described for first time in Mexican children that -844 G/A polymorphism contribute to a significant increase in subcutaneous fat, increasing the risk of developing obesity (OR, 2.6; *p *= 0.01) in children who are carriers of the G/A and A/A genotypes. A possible explanation for this finding could be that the -844 G/A polymorphism contribute to the large amount of PAI-1 produced by adipose tissue expansion, as well as the increase of obesity. Studies of *PAI-1 *knockout mice have shown an effect of PAI-1 on weight gain and increased adipose cellularity associated with high-fat dieting [35]. Besides, studies in which the *PAI-1 *gene was disrupted in ob/ob mice show a reduction of adiposity in these mice. This suggests that *PAI-1 *gene can control fat mass, although the mechanism of action is not yet known, may be *PAI-1 *gene can control fat mass at least in part, by inducing the proliferation of adipocytes through the effect on the expression of genes such as tumour necrosis factor alpha (TNF-α) and transforming growth factor beta (TGF-β), leptin and insulin [36].

In addition the effect on the increase in adipose tissue, there was an association of G/A and A/A genotypes of -844 G/A polymorphism with increased triglyceride levels and decreased HDL-C levels, which indicates that those children who are carriers of these genotypes, have an increase in the risk to develop atherogenic dyslipidemia compared with genotype G/G. In the case of *HindIII *C/G polymorphism, C/G and G/G genotypes were associated with a raise of total cholesterol explaining 8% of the variability of their plasma concentration, influencing along with -844 G/A polymorphism to the development of atherogenic profile that characterizes the MetS. It has been reported that a very-low-density lipoprotein (VLDL)-responsive element in the *PAI-1 *promoter could be responsible for the effect of plasma lipids on PAI-1 expression [14]. Therefore, the increase in PAI-1 levels may contribute to the development of obesity and atherogenic dyslipidemia, and PAI-1 may be a causal link between obesity and cardiovascular disease.

The -844 G/A and *HindIII *C/G *PAI-1 *single nucleotide polymorphisms have not been associated with PAI-1 levels. Several adult studies showed that an increase in the level of PAI-1 was related to the genotype *PAI-1 *4 G/5 G polymorphism [37,38]. However, in children some information is available on the influence of the 4 G/5 G polymorphism on PAI-1 levels or with others obesity-related phenotypes. In Children with obesity, Estelles et al. [39] observed no influence of the 4 G/5 G polymorphism on PAI-1 levels. Moreover, no influence of the *PAI-1 *4 G/5 G polymorphism on lipid and glucose metabolism parameters was observed in Turkish obese children [40,41].

A limitation of this study is the small number of sample, even though is a sample with children that were recruited with precise selection criteria and the control group did not have any of the components included in the definition of MetS. In addition, few studies of genetic association of *PAI-1 *gene with MetS have been conducted in children. Other limitation of this study is that lack of replication, the replication of genetic associations in independent populations is essential to reduce the number of false-positive results and to further define the role of these variants in the susceptibility to complex disease as MetS.

Although our study found an association of -844 G/A polymorphism with the MetS and its components such as obesity and a atherogenic dyslipidemia characterized by hypertriglyceridemia and low HDL-cholesterol, and the *HindIII *C/G polymorphism with increased plasma levels of total cholesterol, other of the limitations is that PAI-1 plasma levels were not measured; therefore the association of -844 G/A and *HindIII *C/G polymorphisms with PAI-1 levels remains uncertain in our population. Therefore it is necessary to determine PAI-1 plasma levels in future studies in Mexican children.

## Conclusions

In summary, this study provide evidence that the -844 G/A *PAI-1 *polymorphism is related with the risk of developing MetS, obesity and atherogenic dyslipidemia, and the *HindIII *C/G *PAI-1 *polymorphism is associated with increased total cholesterol levels, which contributes to the pathogenesis of MetS.

## Abbreviations

MetS: metabolic syndrome; PAI-1: plasminogen activator inhibitor-1; PAS: plasminogen activation system; Pas: physiological plasminogen activators; HDL: high density lipoprotein; 3'UTR: 3'untranslated region; BP: blood pressure; SBP: systolic blood pressure; DBP: diastolic blood pressure; LDL: low density lipoprotein; HOMA-IR: homeostasis model assessment of insulin resistance; gDNA: genomic DNA; PCR-RFLP: polymerase chain reaction-restriction fragment length polymorphism

## Competing interests

The authors declare that they have no competing interests.

## Authors' contributions

IPR conceived and organized the study and prepared the manuscript. UDCM performed the genotyping and participated in the statistical analysis, interpreted the data and wrote the manuscript. LSG and AGC were responsible for patient enrollment and participated in the genetic analysis. JFMV, BIA and ECS participated in the design of the study, contributed interpreting the data and revising successive drafts of the manuscript. All authors read and approved the final manuscript.

## Pre-publication history

The pre-publication history for this paper can be accessed here:

http://www.biomedcentral.com/1471-2431/12/41/prepub

## References

[B1] Huang PL A comprehensive definition for metabolic syndrome Dis Model Mech 2009 2 231 237 10.1242/dmm.001180 19407331 PMC2675814

[B2] Taslim S Tai ES The relevance of the metabolic syndrome Ann Acad Med Singap 2009 38 29 25 19221668

[B3] Alberti KGMM Zimmet P Shaw J Metabolic syndrome--a new world-wide definition. A Consensus Statement from the International Diabetes Federation Diabet Med 2006 23 469 480 16681555 10.1111/j.1464-5491.2006.01858.x

[B4] Bruce KD Byrne CD The metabolic syndrome: common origins of a multifactorial disorder Postgrad Med J 2009 85 614 621 10.1136/pgmj.2008.078014 19892897

[B5] Hamsten A de Faire U Walldius G Dahlén G Szamosi A Landou C Blombäck M Wiman B Plasminogen activator inhibitor in plasma: risk factor for recurrent myocardial infarction Lancet 1987 2 3 9 2885513 10.1016/s0140-6736(87)93050-9

[B6] Juhan-Vague I Thompson SG Jespersen J Involvement of the hemostatic system in the insulin resistance syndrome. A study of 1500 patients with angina pectoris. The ECAT Angina Pectoris Study Group Arterioscler Thromb 1993 13 1865 1873 10.1161/01.ATV.13.12.1865 8241109

[B7] Aso Y Plasminogen activator inhibitor (PAI)-1 in vascular inflammation and thrombosis Front Biosci 2007 12 2957 2966 10.2741/2285 17485272

[B8] Schneiderman J Sawdey MS Keeton MR Bordin GM Bernstein EF Dilley RB Loskutoff DJ Increased type 1 plasminogen activator inhibitor gene expression in atherosclerotic human arteries Proc Natl Acad Sci USA 1992 89 6998 7002 10.1073/pnas.89.15.6998 1495992 PMC49632

[B9] Sobel BE Increased plasminogen activator inhibitor-1 and vasculopathy. A reconcilable paradox Circulation 1999 99 2496 2498 10330378 10.1161/01.cir.99.19.2496

[B10] Morange PE Lijnen HR Alessi MC Kopp F Collen D Juhan-Vague I Influence of PAI-1 on adipose tissue growth and metabolic parameters in a murine model of diet-induced obesity Arterioscler Thromb Vasc Biol 2000 20 1150 1154 10.1161/01.ATV.20.4.1150 10764686

[B11] Naran NH Chetty N Crowther NJ The influence of metabolic syndrome components on plasma PAI-1 concentrations is modified by the PAI-1 4 G/5G genotype and ethnicity Atherosclerosis 2008 196 155 163 10.1016/j.atherosclerosis.2007.03.024 17467713

[B12] Bouchard L Vohl MC Lebel S Hould FS Marceau P Bergeron J Pérusse L Mauriège P Contribution of genetic and metabolic syndrome to omental adipose tissue PAI-1 gene mRNA and plasma levels in obesity Obes Surg 2010 20 492 499 10.1007/s11695-010-0079-1 20127289

[B13] Ha H Oh EY Lee HB The role of plasminogen activator inhibitor 1 in renal and cardiovascular diseases Nat Rev Nephrol 2009 5 203 211 10.1038/nrneph.2009.15 19322185

[B14] Binder BR Christ G Gruber F Grubic N Hufnagl P Krebs M Mihaly J Prager GW Plasminogen activator inhibitor 1: physiological and pathophysiological roles News Physiol Sci 2002 17 56 61 11909993 10.1152/nips.01369.2001

[B15] Ma Z Paek D Oh CK Plasminogen activator inhibitor-1 and asthma: role in the pathogenesis and molecular regulation Clin Exp Allergy 2009 39 1136 1144 10.1111/j.1365-2222.2009.03272.x 19438580

[B16] Arya R Blangero J Williams K Almasy L Dyer TD Leach RJ O'Connell P Stern MP Duggirala R Factors of insulin resistance syndrome-related phenotypes are linked to genetic locations on chromosomes 6 and 7 in nondiabetic mexican-americans Diabetes 2002 51 841 847 10.2337/diabetes.51.3.841 11872689

[B17] Duggirala R Blangero J Almasy L Dyer TD Williams KL Leach RJ O'Connell P Stern MP A major susceptibility locus influencing plasma triglyceride concentrations is located on chromosome 15q in Mexican Americans Am J Hum Genet 2000 66 1237 1245 10.1086/302849 10729112 PMC1288191

[B18] Adamski MG Turaj W Slowik A Wloch-Kopec D Wolkow P Szczudlik A A-G-4 G haplotype of PAI-1 gene polymorphisms -844 G/A, *HindIII *G/C, and -675 4 G/5G is associated with increased risk of ischemic stroke caused by small vessel disease Acta Neurol Scand 2009 120 94 100 10.1111/j.1600-0404.2008.01127.x 19154538

[B19] Lopes C Dina C Durand E Froguel P PAI-1 polymorphisms modulate phenotypes associated with the metabolic syndrome in obese and diabetic Caucasian population Diabetologia 2003 46 1284 1290 10.1007/s00125-003-1170-0 12856128

[B20] Torres-Carrillo NM Torres-Carrillo N Vázquez-Del Mercado M Delgado-Rizo V Oregón-Romero E Parra-Rojas I Muñoz-Valle JF The -844 G/A PAI-1 polymorphism is associated with mRNA expression in rheumatoid arthritis Rheumatol Int 2008 28 355 360 10.1007/s00296-007-0453-z 17899094

[B21] Padilla-Gutiérrez JR Palafox-Sánchez CA Valle Y Orozco-Barocio G Oregón-Romero E Vázquez-Del Mercado M Rangel-Villalobos H Llamas-Covarrubias MA Muñoz-Valle JF Plasminogen activator inhibitor-1 polymorphisms (-844 G > A and *HindIII *C > G) in systemic lupus erythematosus: association with clinical variables Clin Exp Med 2011 11 11 17 10.1007/s10238-010-0099-0 20567875

[B22] Dawson S Hamsten A Wiman B Henney A Humphries S Genetic variation at the plasminogen activator inhibitor-1 locus is associated with altered levels of plasma plasminogen activator inhibitor-1 activity Arterioscler Thromb 1991 11 183 190 10.1161/01.ATV.11.1.183 1670989

[B23] National High Blood Pressure Education Program Working Group on High Blood Pressure in Children and Adolescents The fourth report on the diagnosis, evaluation, and treatment of high blood pressure in children and adolescents Pediatrics 2004 114 555 576 15286277

[B24] Centers for Disease Control and Prevention, National Center for Health Statistics Clinical Growth Charts http://www.cdc.gov/growthcharts/clinical_charts.htm

[B25] Zimmet P Alberti G Kaufman F Tajima N Arslanian S Wong G Bennett P Shaw J Caprio S International diabetes federation task force on epidemiology and prevention of diabetes: the metabolic syndrome in children and adolescents Lancet 2007 369 2059 2061 10.1016/S0140-6736(07)60958-1 17586288

[B26] Miller SA Dykes DD Polesky HF A simple salting out procedure for extracting DNA from human nucleated cells Nucleic Acids Res 1988 16 1215 10.1093/nar/16.3.1215 3344216 PMC334765

[B27] Henry M Chomiki N Scarabin PY Alessi MC Peiretti F Arveiler D Ferrières J Evans A Amouyel P Poirier O Cambien F Juhan-Vague I Five frequent polymorphisms of the PAI-1 gene: lack of association between genotypes, PAI activity, and triglyceride levels in a healthy population Arterioscler Thromb Vasc Biol 1997 17 851 858 10.1161/01.ATV.17.5.851 9157947

[B28] Grenett HE Khan N Jiang W Booyse FM Identification of the Hind III polymorphic site in the PAI-1 gene: analysis of the PAI-1 Hind III polymorphism by PCR Genet Test 2000 4 65 68 10.1089/109065700316507 10794364

[B29] Benza RL Grenett H Li XN Reeder VC Brown SL Go RC Hanson KA Perry GJ Holman WL McGiffin DC Kirk KA Booyse FM Gene Polymorphisms for PAI-1 Are Associated with the Angiographic Extent of Coronary Artery Disease J Thromb Thrombolysis 1998 5 143 150 10.1023/A:1008882113177 10767109

[B30] Torres-Carrillo N Magdalena Torres-Carrillo N Vázquez-Del Mercado M Rangel-Villalobos H Parra-Rojas I Sánchez-Enríquez S Francisco Muñoz-Valle J Distribution of -844 G/A and Hind III C/G PAI-1 polymorphisms and plasma PAI-1 levels in Mexican subjects: comparison of frequencies between populations Clin Appl Thromb Hemost 2008 14 220 226 10.1177/1076029607304747 18263635

[B31] Rubi-Castellanos R Martínez-Cortés G Muñoz-Valle JF González-Martín A Cerda-Flores RM Anaya-Palafox M Rangel-Villalobos H Pre-Hispanic Mesoamerican demography approximates the present-day ancestry of Mestizos throughout the territory of Mexico Am J Phys Anthropol 2009 139 284 294 10.1002/ajpa.20980 19140185

[B32] Grubic N Stegnar M Peternel P Kaider A Binder BR A novel G/A and the 4 G/5G polymorphism within the promoter of the plasminogen activator inhibitor-1 gene in patients with deep vein thrombosis Thromb Res 1996 84 431 443 10.1016/S0049-3848(96)00211-3 8987164

[B33] Henry M Chomiki N Scarabin PY Alessi MC Peiretti F Arveiler D Ferrières J Evans A Amouyel P Poirier O Cambien F Juhan-Vague I Five frequent polymorphisms of the PAI-1 gene: lack of association between genotypes, PAI activity, and triglyceride levels in a healthy population Arterioscler Thromb Vasc Biol 1997 17 851 858 10.1161/01.ATV.17.5.851 9157947

[B34] Torres-Carrillo N Torres-Carrillo NM Martínez-Bonilla GE Vázquez-Del Mercado M Palafox-Sánchez CA Oregón-Romero E Bernard-Medina AG Rangel-Villalobos H Muñoz-Valle JF Plasminogen activator inhibitor-1 C/G polymorphism in relation to plasma levels in rheumatoid arthritis Clin Exp Med 2009 9 223 228 10.1007/s10238-009-0038-0 19238514

[B35] Morange PE Lijnen HR Alessi MC Kopp F Collen D Juhan-Vague I Influence of PAI-1 on adipose tissue growth and metabolic parameters in a murine model of diet-induced obesity Arterioscler Thromb Vasc Biol 2000 20 1150 1154 10.1161/01.ATV.20.4.1150 10764686

[B36] Schäfer K Fujisawa K Konstantinides S Loskutoff DJ Disruption of the plasminogen activator inhibitor 1 gene reduces the adiposity and improves the metabolic profile of genetically obese and diabetic ob/ob mice FASEB J 2001 15 1840 1842 11481248 10.1096/fj.00-0750fje

[B37] Hoffstedt J Andersson LL Persson L Isaksson B Arner P The common -675 4 G/5G polymorphism in the plasminogen activator inhibitor-1 gene is strongly associated with obesity Diabetologia 2002 45 584 587 10.1007/s00125-001-0774-5 12032637

[B38] Sartori MT Vettor R De Pergola G De Mitrio V Saggiorato G Della Mea PD Patrassi GM Lombardi AM Fabris R Girolami A Role of the 4 G/5G polymorphism of PAI-1 gene promoter on PAI-1 levels in obese patients: influence of fat distribution and insulin-resistance Thromb Haemost 2001 86 1161 1169 11816701

[B39] Estelles A Dalmau J Falco C Berbel O Castello R Espana F Aznar J Plasma PAI-1 levels in obese children-effect of weight loss and influence of PAI-1 promoter 4 G/5G genotype Thromb Haemost 2001 86 647 652 11522017

[B40] Kinik ST Ataç FB Verdi H Cetintaş S Sahin FI Ozbek N The effect of plasminogen activator inhibitor-1 gene 4 G/5G polymorphism on glucose and lipid metabolisms in Turkish obese children Clin Endocrinol 2005 62 607 610 10.1111/j.1365-2265.2005.02268.x 15853833

[B41] Kinik ST Ozbek N Yuce M Yazici AC Verdi H Ataç FB PAI-1 gene 4 G/5G polymorphism, cytokine levels and their relations with metabolic parameters in obese children Thromb Haemost 2008 99 352 356 18278185 10.1160/TH07-06-0395

